# Computational Modelling of VIG Plates Using FEM: Static and Dynamic Analysis

**DOI:** 10.3390/ma15041467

**Published:** 2022-02-16

**Authors:** Izabela Kowalczyk, Damian Kozanecki, Sylwia Krasoń, Martyna Rabenda

**Affiliations:** 1 Department of Structural Mechanics, Lodz University of Technology, Politechniki 6, 93-590 Lodz, Poland; 238124@edu.p.lodz.pl (I.K.); 238125@edu.p.lodz.pl (D.K.); 238126@edu.p.lodz.pl (S.K.); 2 Department of Concrete Structures, Lodz University of Technology, Politechniki 6, 93-590 Lodz, Poland

**Keywords:** plate, finite element method, dynamic analysis, static analysis, vacuum insulated glass

## Abstract

Vacuum insulated glass (VIG) panels are becoming more and more popular due to their good thermal performance. Little information about the mechanical or strength parameters, which are crucial for the durability of a window, might be found in the published papers. The purpose of this work was to analyse the impact of different parameters on VIG panels’ mechanical properties. Parameter diversity refers to both geometrical and material characteristics. Static and dynamic analyses using the finite element method (ABAQUS program) were conducted. In addition, 101 various numerical models, created with the use of Python language, were tested. The changes of geometrical parameters were made with constant material parameters and the reverse. It has been concluded that pillars’ material and geometrical properties are crucial considering not only the static response of the VIG plates, but also the dynamic one. Moreover, it was proven that getting rid of the first row of pillars near every edge seal led to an increase in deflection of the glass panes. Additionally, considering results for dynamic response associated with out-of-phase vibrations, spacing between support pillars should be large enough in order to avoid possible damage to the glass pane due to rapidly decreasing distance between them. Further research opportunities have been described.

## 1. Introduction

### 1.1. Research Significance

Nowadays, energy consumption is one of the most urgent issues in most countries of the world. In ref [1], a consistent and comprehensive source of information on thermal energy use in buildings was given. Past, present, and future trends on a global and regional basis were also shown. Additionally, the buildings, which form a large part of global and regional energy demand (32% of global final energy demand, 30% of energy-related CO_2_ emissions), were presented. Furthermore, the energy consumed for space heating and cooling is 40% of the total energy demand in commercial buildings and 61% in residential buildings. The information included in the paper [1] confirm that construction plays a large role in global and regional energy consumption.

**Figure 1:**
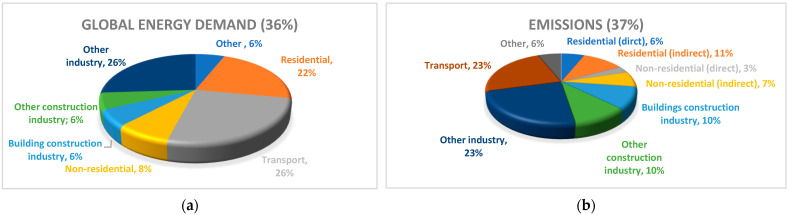
(**a**) Global share of buildings and construction final energy; (**b**) buildings and construction’s share of global energy-related CO_2_ emissions; 2020 [2].

Furthermore, the newest report of International Energy Agency (IEA) states that, since the signing of the Paris Agreement in 2015, greenhouse gas emissions from the buildings and construction sector have peaked (in 2019) and subsequently fallen to 2007 levels [2]. The latest drop was due to the COVID-19 pandemic that impacted global economy (reduction in energy demand), which masks the reality that the decarbonization of the buildings is not on track. If the COVID-19 effects are removed, the decarbonization level in 2020 is only 40% of the 2050 reference path goal. In summary, buildings accounted for 36% of global energy demand (Figure 1) and 37% of energy-related CO_2_ emissions in 2020.

In order to achieve the Agreement’s goal, the built environment must be decarbonized throughout its life cycle and simultaneously the resilience of buildings should be increased. To do this, three aspects should be considered: reduction of energy demand and increase of energy efficiency, and decarbonization of the energy system, and solution of the embodied carbon stored in building materials [2]. According to the report [2], many initiatives must be delivered so that greenhouse gas emissions will be reduced. One of the main steps is to upgrade the building envelope, which plays an important role in monitoring building energy by adjusting the cooling/heating loads between outdoor and indoor environments.

In this regard, in recent studies, improvement solutions, which focus on the techniques and methods, are implemented. In ref [3], the importance of phase change materials (PCMs) applicated in roof and external walls, which positively influence the thermal comfort and building saving energy, is highlighted. Additionally, to minimize the energy and resources consumption due to the construction, the concrete structures should use the material in the most efficient way considering its strength and durability within the service life of the structure. There are many works investigating this issue. In ref [4], both green concrete and ultra-high-performance concrete are presented. It has been pointed out that the sustainability potential of building materials and whole buildings or structures are complex. Three issues should be considered: environmental impact, technical performance, and lifetime. In ref [5], new ecological concrete with CO_2_ emission below zero is described. Going forward, Ferrari et al. [6] showed a new technology used for transforming returned concrete into a granular material, which may be reused as aggregate for concrete. This new technology has environmental, social, and economic advantage.

A review of data-driven techniques for building energy consumption modelling and forecasting is performed in [7]. Those techniques are presented also in [8,9], where machine learning algorithms and artificial neural networks are used for building energy prediction. The application of the techniques described in the above literature positions allows for achieving relatively high forecasting accuracy, which is crucial for finding areas whose improvement will have a positive impact on energy consumption. Considering all written above, the energy consumption is a high-priority issue around which various studies are conducted. In this paper, one of the building components, which is a thermal weak point in building the envelope, will be presented.

Currently, commonly used glazing in buildings is characterized by much worse insulation parameters than other building partitions [10]. Therefore, they provide a pathway for significant heat transfer between the indoor and outdoor environments. Traditional window technology involves two or more panes of glass separated by a space filled with air or gas. Vacuum Insulated Glazing (VIG)—composite window in which the space between the glass panes is insulated with a vacuum—is one of the most advanced technologies allowing a significant reduction in the energy loss of a building. In windows where there is no air or gas between the panes, conduction and convection can be minimized [11].

Vacuum glazing technology was described in the patent literature as early as 1913 by Zoller. Since that time, many proposals for vacuum glazing designs have been reported, largely concerned with the requirements for maintaining separation between glazing or the need for edge sealing. However, it was not until 1989 that the first successful production of flat vacuum glazing was reported and presented by Collins at the ISES congress. The specimen produced at that time consisted of two sheets of soda-lime glass separated by a series of pillars, on the edges of which was fused brazed glass. Air was pumped out by means of holes in the glass sheet. The first VIG plate was made by hand [12]. A heat transfer coefficient of 0.8 W/m^2^ K was obtained in the central region of the vacuum glass sample [13].

**Figure 2:**
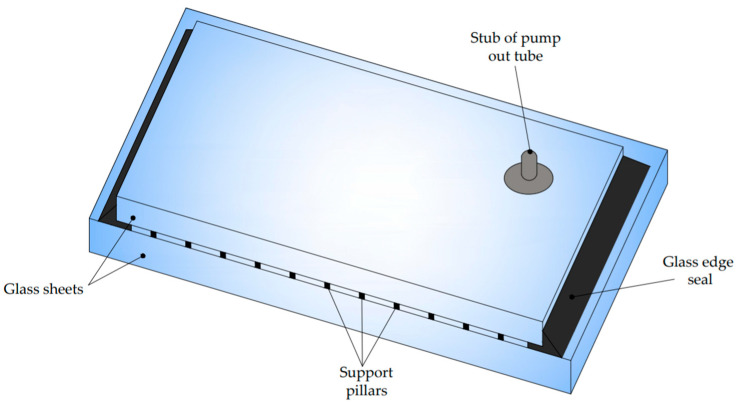
Scheme of construction of the VIG unit.

At present, the production process of VIG-type panels is automated, and the panels themselves are widely used in construction, transportation, and household appliances. The heat transfer coefficient can be as low as 0.4 W/m^2^ K. The construction of the board has also changed slightly, the diagram of which is shown in Figure 2.

As may be noted, vacuum glazing consists of two panes of glass hermetically sealed around the edges with a vacuum gap between the panes. However, due to the significant stresses resulting from atmospheric pressure, high strength tempered glass is now used [14].

To prevent collapse and contacting of the glass panes under atmospheric pressure, an arrangement of support pillars is also placed between the glass panes. Wherein the pillars comprise a body and a functional layer on at least a portion of the body, the diameter of the pillars is equal to or less than 600 micrometres, and the compressive strength of the pillars is equal to or greater than 400 MPa [15]. They are usually made of metal, which makes them a thermal bridge. However, reducing the size or number will result in higher stress levels in the glass due to contact and bending of the glass. An alternative to metal is a high mechanical strength ceramic or glass material, which have significantly better thermal performance. The contact stresses at the tap of the glass panes are reduced with a surface coating [15].

Currently, commonly used solder material as an edge seal is a rigid edge seal. There is a proven method that allows for repeatability and durability with respect to the hermetic edge seal. The use of edge seals without brazing glass is also being considered [15].

In this paper, finite element modelling of VIG plate was performed. Nowadays, this technique is widely used to analyse many research concerns. Teotia and Soni [16] used finite element modelling for complex damage behaviour of a sandwiched polymer composite (laminated glass). The experimental and simulation results of the flexural behaviour of the three different laminated glass plates were compared in the work [17]. In ref [18], the dynamic characterisation of an existing glass suspension footbridge is presented and later an FEM is performed, in order to additionally assess and explore the structure performance, including a sensitivity investigation. Regarding VIG plates, FEM was used, inter alia, for investigating the effects of various pillar design parameters (thermal conductivity, geometry, and arrangement) on the VIG thermal performance [19].

The use of advanced fractal methods to analyse the distribution of pillars inside the VIG structure was also considered. These methods are now successfully used in many fields of engineering, from mechanics [20] to diffusion issues [21]. Depending on the analysed problems, fractal dimension of the structure is calculated by the Minkowski–Bouligand (box-counting) method [22] or mass–radius method [23]. In many applications, the connection of the fractal dimension to certain properties of the analysed structures allowed the authors to provide a more in-depth and extended substantive analysis [24].

In the presented research, due to the periodicity of pillars distribution and the fact that the main purpose of this work is to analyse not geometric but material properties of the structure, direct use of fractal methods was omitted. In the case of problems related to the optimization of pillars arrangement inside the VIG structure, the application of fractal methods is considered.

### 1.2. Motivation

The excellent insulating properties of VIG are reflected in numerous studies. Cho and Kim tested the heat transfer coefficient of vacuum glazing through simulation and physical testing [25], while Ashmore, Cabrera, and Kocer investigated the acoustic properties of vacuum glazing [26]. However, it is much more difficult to find information on the mechanical or strength parameters, which are crucial for the durability of a window. Zhu considered the effects of pillars and sealing design on thermal and mechanical performance of Vacuum Insulated Glazing (VIG). In his research, the effects of various design parameters of these element were investigated through the finite element method (FEM), along with experimental validation and analytical calculation. In mechanical investigation, he checked the impact of the temperature on displacement and compared maximum shear stress for different types of sealing [27]. The analyses carried out by us will allow not only to extend the current knowledge about this innovative product but will also make it possible to apply them more widely. VIG panels are exposed to constant stress caused by atmospheric pressure and temperature differentials which may lead to damage panels and edge seal. Currently, there is no possibility to verify VIG components properties without damaging the panels. Considering that, non-destructive character of our research is desired and allows for conducting any number of simulations.

### 1.3. Purpose and Scope of Work

In this study, static and dynamic analyses were carried out to examine an impact of different parameters on VIG panels’ mechanical properties. In the case of VIG units, the changes in Young’s modulus for the sealing are irrelevant and, for the glass, it is impossible. The impact of Young’s modulus for the pillars is crucial because this affects deflection and wear issues over time. The influence of external factors such as UV radiation is crucial to the durability of many materials during their use. While it does not affect the glass, it can affect the material properties of the pillars, which should not change over time. When glazing buildings, optical and visual properties are also an important consideration. Therefore, the arrangement, size and material of pillars in VIG panels must take these into account. Parameter changes refer to geometrical (spacing and number of support-pillars, thickness of glass panes, pillar diameter) and material characteristics (Young’s modulus of support-pillars and seal). There were 101 various numerical models tested. The changes of material parameters were made with constant geometrical parameters and vice versa. The results of static experiments were presented as maximum displacements of glass panes under atmospheric pressure, whereas the dynamic results were gathered as natural frequencies for each model. The results for both types of analyses were compiled and compared for variable parameters.

## 2. Materials and Methods

### 2.1. Finite Element Modelling—Introduction

In the presented research, the finite element method (ABAQUS program) was used to perform static and dynamics analyses. Deflections of the tested glass plates under assumed loads (atmospheric pressure) are tiny and analyses are limited to the elastic range of the material behaviour. Under these assumptions, FEM provides proper results. Furthermore, FEM allowed for creating a model with full 3D representation of support pillars, and consequently provide greater accuracy [27,28]. The adopted model allows for including the stress concentration in support pillars and shear deformation. In Section 2.2, the selection of the mesh size was analysed. It was shown that the proper selection of the mesh within the pillar provides a better representation of its deformation nature.

Calculations in ABAQUS/CAE 2017 were performed using PLGrid Infrastructure, which provides access to clusters located in five High Performance Computing centres. Computational tasks are outsourced through a so-called middleware, which manages the resources of all hubs centrally. The WCSS hub was used in this analysis. CPU run time was about 12–20 min and memory requirements about 650 MB for every separate model.

The model was created as 3D solid extrusion. Each part of the model (pillars, sealing and glasses) was created separately. The materials for each part were assumed as elastic, which determines to introduce Young’s modulus and Poisson’s Ratio. Furthermore, the material density was set, which is needed for dynamic analysis. In order to connect all the parts, a tie constraint has been used, where the master and slave surfaces are assigned for pillars/sealing and glass, respectively. To achieve the best possible contact simulation, the following selection of the master/slave surface was chosen. The slave surface was assumed to be the more finely meshed.

**Figure 3:**
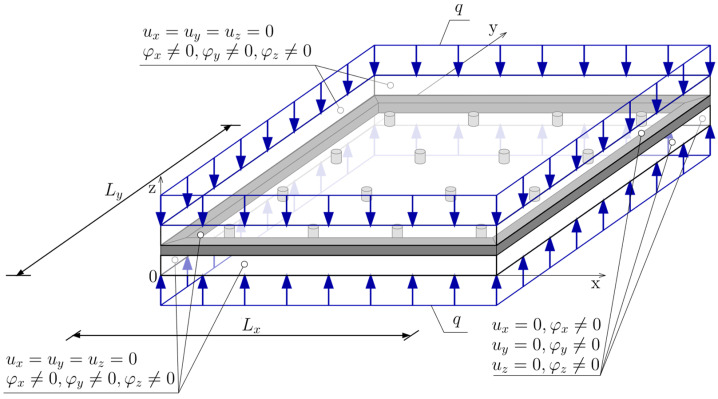
VIG plate computational model—boundary conditions and loads.

The static scheme of the considered VIG plate was assumed as the plate fixed at the edges. Boundary conditions were represented in the FEM model as fixed displacements along axes *x*, *y*, and *z* (*u_x_*, *u_y_* and *u_z_*) on the side surfaces of elements imitating glazing, but rotation degrees of freedom (*φ*_x_, *φ_y_* and *φ_z_*) remain free (Figure 3). Nevertheless, the rotation on the edges in the model hardly occurs, since supports cover the entire side surface, not just the linear edge. To simulate the vacuum between two glass panes, a surface load (*q*) was applied on the external surfaces of solid elements imitating glass. The load value is approximate to atmospheric pressure and equals 0.1 MPa.

**Figure 4:**
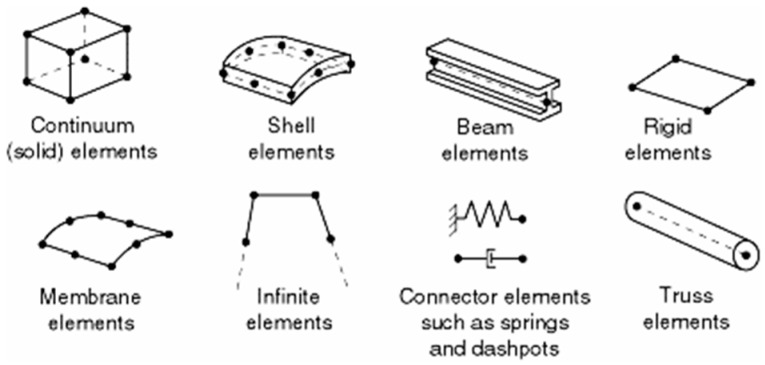
Commonly used element families in ABAQUS [29].

Next, all the parts have been meshed. The meshing procedure was shown in Section 2.2. There are five factors that characterize elements in ABAQUS: family, degrees of freedom (related to the element family), number of nodes, formulation, and integration. Due to that, the element obtains a name that identifies each of the factors. Commonly used element families are shown in Figure 4.

**Figure 5:**
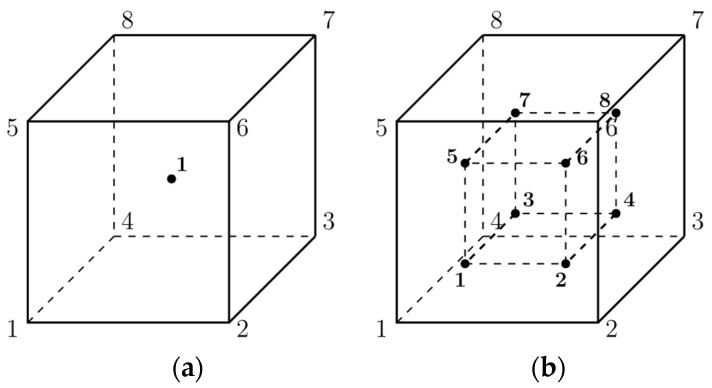
(**a**) C3D8R element with reduced integration (1 integration point) versus (**b**) C3D8 element (fully integrated—2 × 2 × 2 integration points) [30].

The degrees of freedom for stress/displacement simulation are the translations and rotations. Regarding the number of nodes and order of the interpolation, displacements are calculated at the nodes of the element and displacements at any other point are obtained by interpolating from the nodal displacement. Commonly, the order of the interpolation is determined by the number of nodes used in elements. For a 3D solid 8-node brick element, there are two types distinguished: C3D8R and C3D8 showed in Figure 5.

**Figure 6:**
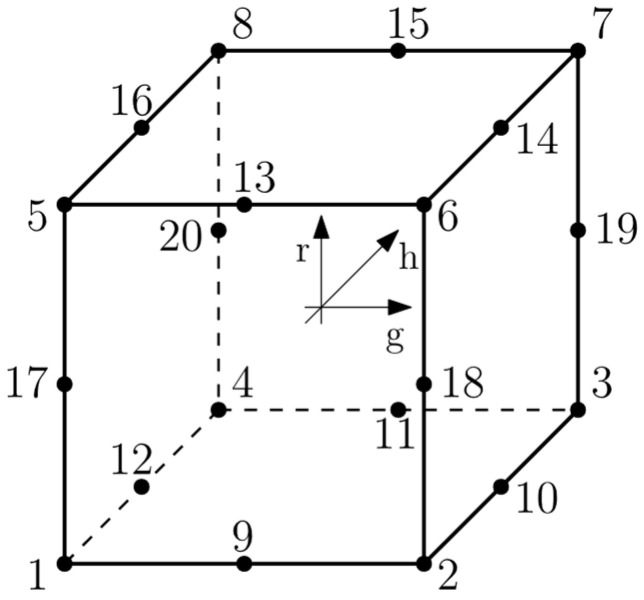
C3D8R element designations [29].

In this paper, C3D8R was assumed (Figure 6)—first order, reduced integration element with hourglass control activated.

The interpolation function ***u*** for C3D8R element is given by Equation (1):

(1) $$u=N^{I}\left(g,h,r\right)u^{I}\;\mathrm{sum}\;\mathrm{on}\;I.$$

where *N^I^* are isoparametric shape functions and ***I*** is the node of the element.

The shape functions are the same as for the C3D8 element and can be found in [29,30] (2):

(2) $$N^{I}\left(g,h,r\right)=\frac{1}{8}\Sigma^{I}+\frac{1}{4}g\Lambda_{1}^{I}+\frac{1}{4}h\Lambda_{2}^{I}+\frac{1}{4}r\Lambda_{3}^{I}+\frac{1}{2}hr\Gamma_{1}^{I}+\frac{1}{2}gr\Gamma_{2}^{I}+\frac{1}{2}gh\Gamma_{3}^{I}+\frac{1}{2}ghr\Gamma_{4}^{I}\;$$

where *I* denotes the node of the element. The last four vectors $\Gamma_{\alpha }^{I}$, α=1,2,3,4 are the hourglass base vectors. The gradient matrix $B^{I}$ is defined by integrating over the element:

(3) $$B_{i}^{I}=\frac{1}{V_{el}}\int_{V_{el}}^{\;}N_{i}^{I}\left(g,h,r\right)dV_{el,\;}N_{i}^{I}\left(g,h,r\right)=\frac{\partial N^{I}}{\partial x_{i}}$$

where $V_{el}$ is volume of the element and *i = 1, 2, 3*. In the centroidal strain formulation, the gradient matrix is simply given:

(4) $$B_{i}^{I}=N_{i}^{I}\left(0,0,0\right)\;$$

Considering the above, it may be seen that centroidal strain formulation reduces the amount of effort required to compute the gradient matrix. In ABAQUS, the artificial stiffness method and the artificial damping method showed in [31] are used to control the hourglass modes in these elements. In ref [32], its effectiveness of C3D8R elements with hourglass control was compared to other types of elements.

The final aspect is element formulation, which refers to the mathematical theory used to define the element’s behaviour. All of the stress/displacement elements in ABAQUS are based on the Lagrangian or material description of behaviour.

The linear static and dynamic analysis of VIG plate has been done in this paper. To perform a dynamic analysis, the linear perturbation step was created. The response in a linear analysis step is the linear perturbation response about the base state (step prior to the linear perturbation step). The frequency extraction performs eigenvalue extraction to calculate the natural frequencies and the corresponding mode shapes of a system. Geometric linearity was accounted for. The eigenvalue problem for the natural frequencies was considered:

(5) $$\left(-\omega^{2}M^{MN}+K^{MN}\right)\phi^{N}=0\;$$

where $M^{MN}$ is the mass matrix, $K^{MN}$ is the stiffness matrix, $\phi^{N}$ is the eigenvector (mode of vibration), and *M* and *N* are degrees of freedom. There are three extraction methods: Lanczos (default method), AMS (Automatic multi-level substructuring) and subspace iteration. The Lanczos method was chosen and a 10 eigenvalues set.

### 2.2. Mesh Density Assumptions

The subjected elements (VIG plates) consist of support pillars that are tiny compared to glass panes. When it comes to choosing a proper global mesh size, the areas of the glass panes and the support pillars contact surfaces have to be carefully taken into account. The mesh size of the glazing needs to be larger than the mesh size of the support pillars in order to maintain the reasonable size of the numerical model. In accordance with ABAQUS documentation [29], the nodes of one finite element, located inside another finite element, are artificially linked by an algorithm to the nodes of the latter.

**Figure 7:**
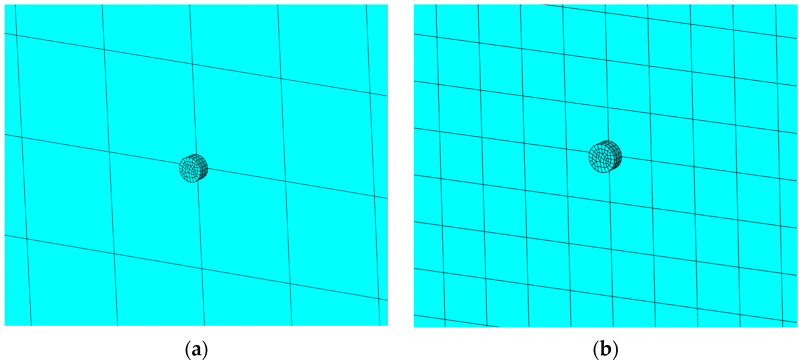

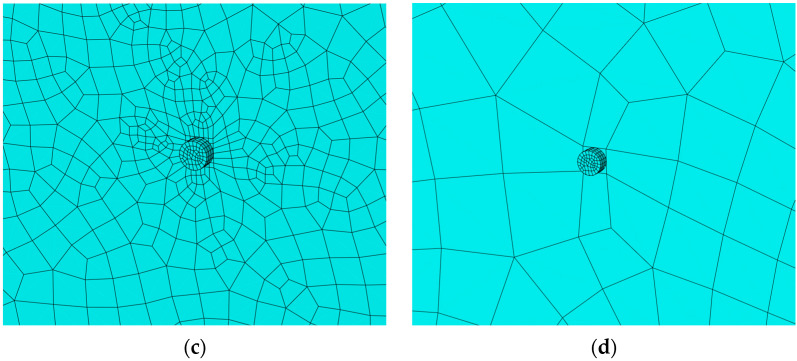
Glass pane mesh near the support pillar for the following mesh sizes: (**a**) 2.5 mm; (**b**) 1.0 mm; (**c**) 1.0 mm (0.1 mm near the support pillars); and (**d**) 2.5 mm (0.5 mm near the support pillars).

When looking for the most accurate numerical solution of the considered problem, the test models were created and several mesh densities assumed. In order to determine first natural frequencies associated with out-of-phase vibrations, the calculations were performed, with the following glass panes’ mesh sizes: 2.5, 1.0, 1.0 (0.1 mm near the support pillars), and 2.5 mm (0.5 mm near the support pillars) (Figure 7).

**Figure 8:**
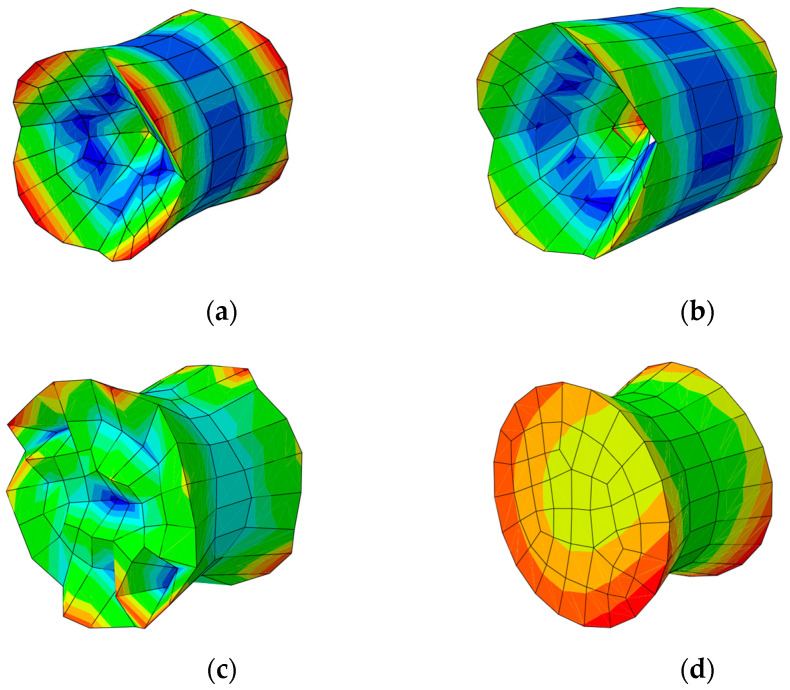
Shape of the support pillar deformation for the following mesh sizes: (**a**) 5.0 mm; (**b**) 1.0 mm; (**c**) 1.0 mm (0.1 mm near the support pillars); and (**d**) 5.0 mm (0.5 mm near the support pillars).

Shapes of the support pillars’ deformations in first natural frequencies associated with out-of-phase vibrations for obtained mesh sizes are shown in Figure 8, respectively.

Determined natural frequencies are 5941.5, 6752.4, 7584.3 and 7634.6 Hz, respectively. The finer the glass pane mesh in the vicinity of the contact surfaces is, the greater the natural frequencies in those cases are.

Comparing the obtained results, creating one tiny finite element under the support pillar is the most effective solution (Figure 7d), which leads to the accurate numerical result and reduces computational costs comparing to assumptions made in Figure 7c. This approach was assumed for the purpose of the further investigation.

### 2.3. Python as a Workflow Optimizer for the ABAQUS Program

In order to effectively create a large number of models with variable parameters, ABAQUS Scripting in Python language (Python 3.9.5 release), as an alternative for graphical user interface in the ABAQUS program, was used. The script that includes all the geometrical and mechanical properties of the subjected elements was created and then implemented. It decisively shortened the time of the numerical models’ preparation.

**Figure 9:**
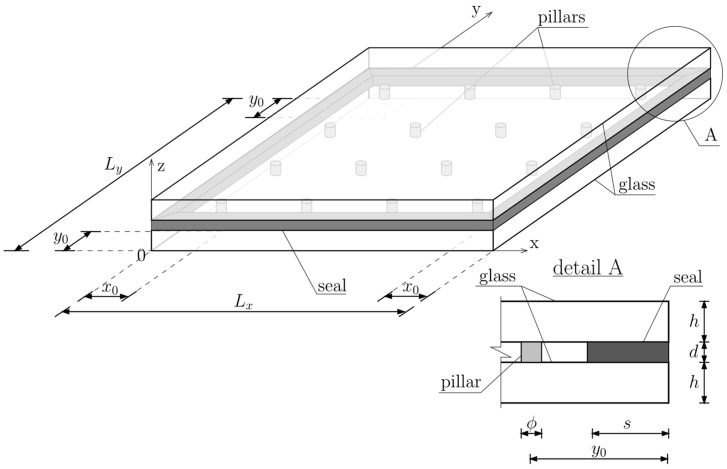
VIG plate computational model—geometry.

The discussed algorithm consists of a class that defines the entire process of creating one computational model of a VIG plate. It contains methods that allow for assigning all geometrical parameters (Figure 9), all material parameters and the meshing methods, also taking into account the parameters of the mesh refinement on the glass panes discussed in the previous Section 2.2. Not only is the main method of the programmed class responsible for creating the entire numerical model based on the values specified in the previous methods, but also for setting the selected support conditions for the elements, determining the amount of the desired modes of free vibrations, and determining the magnitude and location of the applied surface load.

Although, for the purpose of the analysis, only selected parameters were assumed as variables (Table 1 and Table 2), the script also allows for changing all the other parameters that were assumed as constants in the further analysis (Section 2.4). Considering the use of advanced fractal methods, there is a possibility to analyse unrestricted non-periodic distribution of the pillars inside the VIG structure or distribution described by slowly varying function, in conjunction with the Python script.

### 2.4. Subject of the Study

The numerical model of the VIG plates used in this study consists of two rectangular elements imitating two glass panes, cylindrical elements between them representing support pillars, and elements imitating seal around the edges of glass (Figure 9).

A series of numerical analyses were carried out and the obtained results were presented in two main categories. The former concerns the change of geometrical parameters, the latter, the change of material properties of glass, pillars, and seal.

The characteristics of seal around the glass panes remain constant except its Young’s modulus. The seal Poisson’s Ratio and density are *ν_s_* = 0.31 and *ρ_s_* = 7850 kg/m^3^, respectively. The width of the seal is 10 mm around the perimeter of the plate, and its thickness is equal to the thickness of the vacuum between the panes—0.3 mm. It was also assumed that, in both categories, the following characteristics of glass and pillars are constant: Young’s modulus of glass *E_g_* = 72 GPa, glass density *ρ_g_* = 2500 kg/m^3^, pillars density *ρ_p_* = 7850 kg/m^3^, Poisson’s Ratio of glass *ν_g_* = 0.22; Poisson’s Ratio of pillars *ν_p_* = 0.31; thickness of vacuum layer *d* = 0.3 mm, dimensions of glass panes—width *L_x_* = 0.60 m and length *L_y_* = 1.20 m.

In the first category of analysis, variable parameters are: glass panes thickness (*h*), coordinates of the extremely located pillar in relation to the edges of the panes (*x*_0_, *y*_0_), number of pillars along the glass edges (*n_x_*, *n_y_*), and diameter of pillars (*ϕ*). Material parameters are constant: Young’s modulus of pillars *E_p_* = 200 GPa, Young’s modulus of edge seal *E_s_* = 200 MPa.

In the second category, the variable parameters are Young’s modules of pillars and edge seal. The other parameters remain constant: glass panes thickness *h* = 5 mm, coordinates of extremely located pillar *x*_0_ = *y*_0_ = 50 mm, number of pillars along the edges *n_x_* = 19, *n_y_* = 39, and diameter of pillars *ϕ* = 0.5 mm.

Values of variable parameters in both categories, geometrical and material properties, are presented in Table 1 and Table 2, respectively.

## 3. Summary and Results

### 3.1. Static Analysis

#### 3.1.1. SA—Analysis of Geometrical Parameters

**Figure 10:**
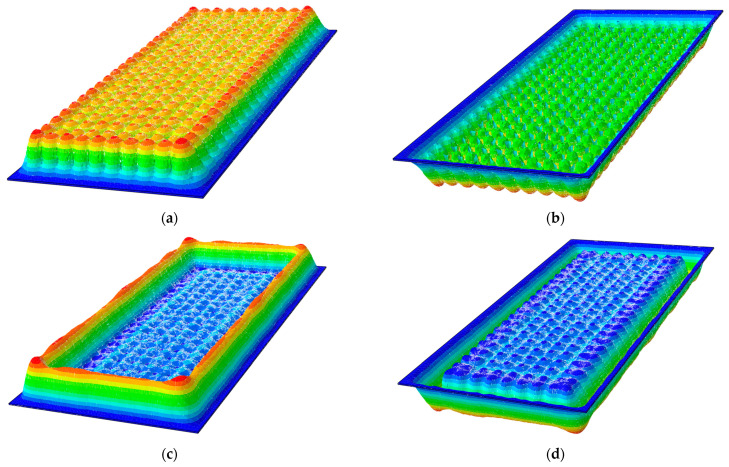
Shapes of the (**a**) bottom and (**b**) top glass panes deformations for *x*_0_ = *y*_0_ = 50 mm as well as (**c**) bottom and (**d**) top glass panes for *x*_0_ = *y*_0_ = 100 mm.

In this section, the analysis of variable geometrical parameters based on a static analysis of the subjected VIG plates has been carried out. Their maximum deflections in relation to a thickness of the glass panes and a diameter of the support pillar have been investigated. Shapes of the bottom and the top glass panes’ deformations are shown in Figure 10.

**Figure 11:**
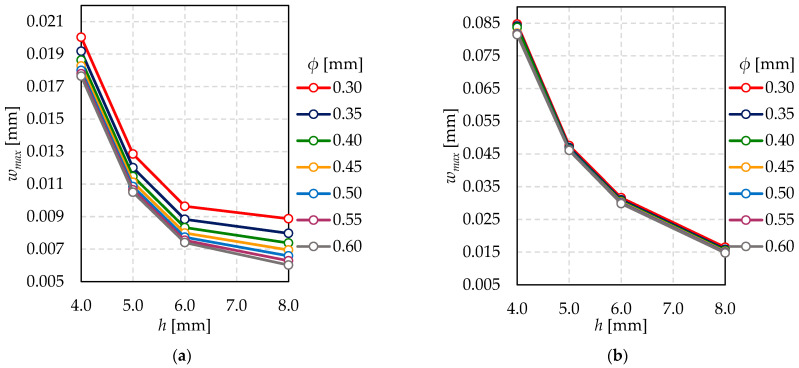
The relationship between maximum glass deflection (*w_max_*) and glass panes thickness (*h*) for different pillar diameter (*ϕ*)—(**a**) *x*_0_ = *y*_0_ = 50 mm; (**b**) *x*_0_ = *y*_0_ = 100 mm.

The pillars’ dimension effects on maximum glass deflection are shown in Figure 11. This influence is more significant for *x*_0_ = *y*_0_ = 50 mm, where the total number of pillars is larger than for *x*_0_ = *y*_0_ = 100 mm. Glass deflection values are greater for cases with a smaller number of pillars. Moreover, the smaller pillar diameter, the sharper maximum deflection growth. Nevertheless, the overall effect of the pillars diameter on maximum deflection is slight—a change in pillars diameter by 0.05 mm causes a change in the deflection up to 10%.

#### 3.1.2. SA—Analysis of Material Parameters

In this section, the analysis of variable material parameters based on a static analysis of the subjected VIG plates has been carried out. Their maximum deflections in relation to Young’s modulus of pillars and Young’s modulus of edge seal has been investigated. Shapes of the bottom and the top glass panes’ deformations are the same as for the analysis presented in Section 3.1.1 (Figure 10).

**Figure 12:**
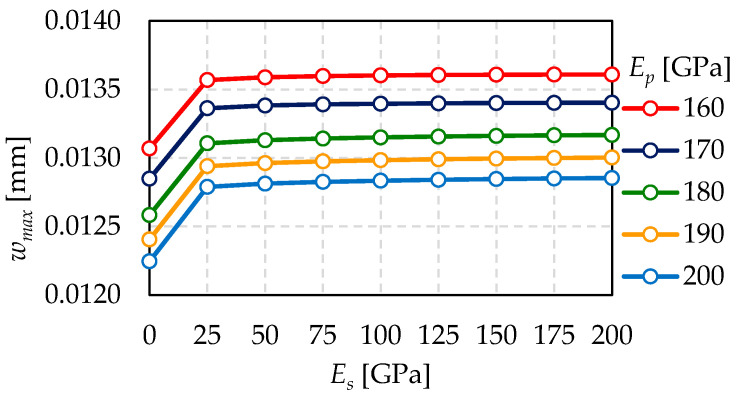
The relationship between maximum glass deflection (*w_max_*) and sealing Young’s modulus (*E_s_*) for different pillar Young’s modulus (*E_p_*).

The obtained results are shown in Figure 12. Maximum deflection of the VIG plate for sealing Young’s modulus *E_s_* = 200 GPa is up to 0.5% higher than the one for sealing Young’s modulus *E_s_* = 25 GPa, considering all the examined *E_p_* values. It is caused by support zone hardening. The greater the sealing of Young’s modulus, the smaller a deflection of a glass pane between a sealing and a support pillar as well as the greater the deflection of a glass pane between the first and the second pillar. The mentioned difference is decisively larger comparing the sealing of Young’s modulus *E_s_* = 0.01 GPa and sealing Young’s modulus *E_s_* = 25 GPa. It is equal to up to 5.0%, considering all the examined *E_p_* values.

Nonetheless, the greater support pillar for Young’s modulus, the lower a maximum glass pane deflection.

As initially expected, the change in material properties of the support-pillars has a much greater impact on the tested properties than the material parameters’ changes of the sealing, which was confirmed in the presented research (Figure 12). Therefore, to provide more accurate results, the 3D model was created. Initially, the adoption of a hybrid model: plate-bar, which was repeatedly used by other researchers, was considered [32]. However, the authors expected a significant influence of the support-pillars properties on the deflections [12]; consequently, the chosen model allows for taking into account the stress concentration in pillars and their shear deformation.

### 3.2. Dynamic Analysis

#### 3.2.1. DA—Analysis of Geometrical Parameters

**Figure 13:**
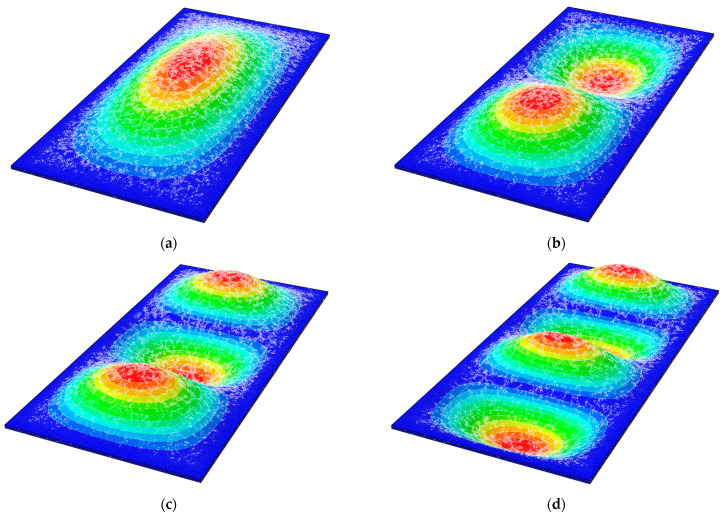
Shape modes for the (**a**) first; (**b**) second; (**c**) third; and (**d**) fourth natural frequencies.

Proceeding to the following calculations, the analysis of variable geometrical parameters based on a free vibration analysis of the subjected VIG plates has been carried out. Their first ten natural frequencies in relation to a thickness of the glass panes and a diameter of the support pillar have been investigated. Mode shapes for the first four natural frequencies are shown in Figure 13.

**Figure 14:**
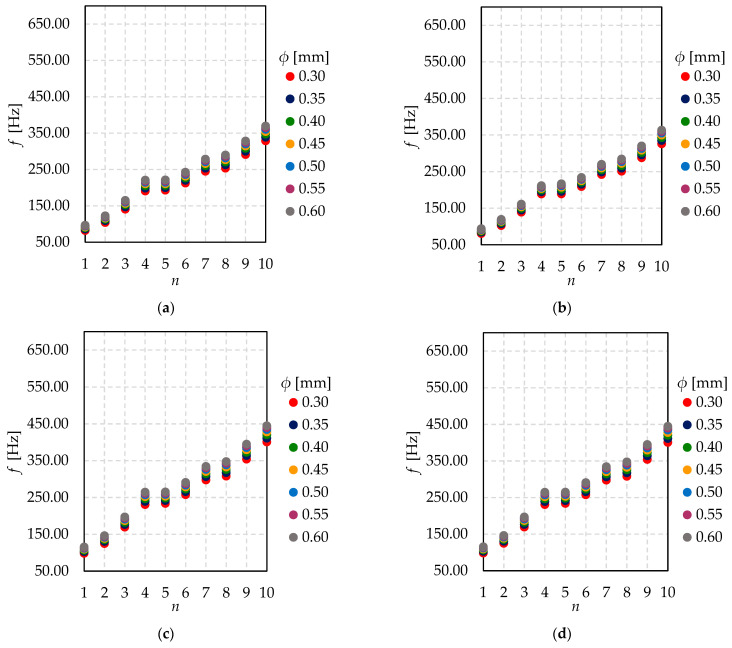

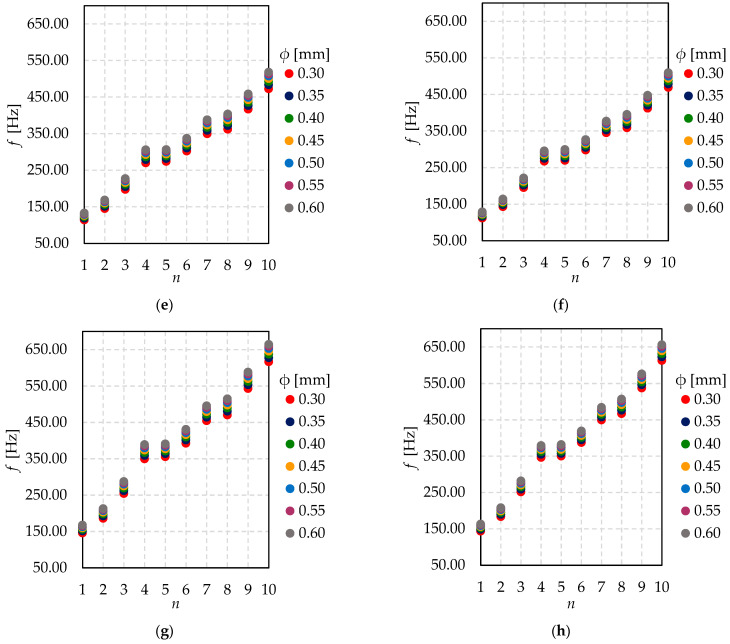
The relationship between natural frequency (*f*) and mode shape number (*n*) for different pillar diameter (*ϕ*)—(**a**) *h* = 4 mm and *x*_0_ = *y*_0_ = 50 mm; (**b**) *h* = 4 mm and *x*_0_ = *y*_0_ = 100 mm; (**c**) *h* = 5 mm and *x*_0_ = *y*_0_ = 50 mm; (**d**) *h* = 5 mm and *x*_0_ = *y*_0_ = 100 mm; (**e**) *h* = 6 mm and *x*_0_ = *y*_0_ = 50 mm; (**f**) *h* = 6 mm and *x*_0_ = *y*_0_ = 100 mm; (**g**) *h* = 8 mm and *x*_0_ = *y*_0_ = 50 mm; (**h**) *h* = 8 mm and *x*_0_ = *y*_0_ = 100 mm.

Figure 14 shows a slight increase of natural frequencies *f_n_* for *n* = 1, 2, …, 10, within the growing value of the pillar diameter. According to the results, the support-pillars number has no influence on the values of the natural frequencies. Moreover, the differences between values of natural frequencies within every next mode shape are more significant for the highest tested mode shapes than for the lowest. Obviously, eigenfrequency values go up as the glass is thicker.

**Figure 15:**
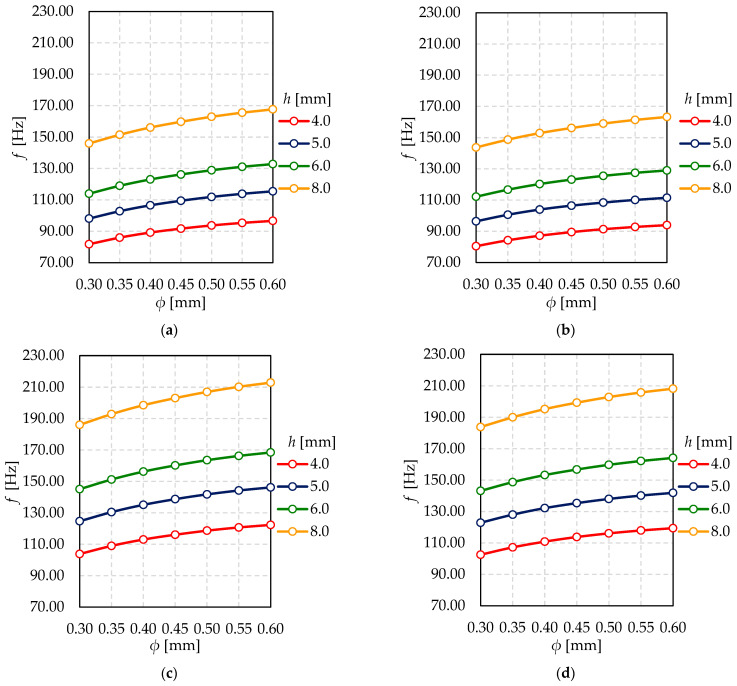
The relationship between natural frequency (*f*) and support pillar diameter (*ϕ*) for different glass thickness—(**a**) mode shape *n* = 1 and *x*_0_ = *y*_0_ = 50 mm; (**b**) mode shape *n* = 1 and *x*_0_ = *y*_0_ = 100 mm; (**c**) mode shape *n* = 2 and *x*_0_ = *y*_0_ = 50 mm; (**d**) mode shape *n* = 2 and *x*_0_ = *y*_0_ = 100 mm.

In Figure 15, the relationship between natural frequency and support pillar diameter for different glass thicknesses is shown. It might be seen that coordinates of extremely located pillar have no influence on natural frequencies. Certainly, together with the growth of the shape mode number, the value of natural frequency increases.

#### 3.2.2. DA—Analysis of Material Parameters

In this section, the analysis of variable geometrical parameters based on a free vibration analysis of the subjected VIG plates was carried out. Their first’ ten natural frequencies in relation to Young’s modulus of pillars and Young’s modulus of edge seal were investigated. Mode shapes for the first four natural frequencies are the same as for the analysis presented in Section 3.2.1 (Figure 13).

**Figure 16:**
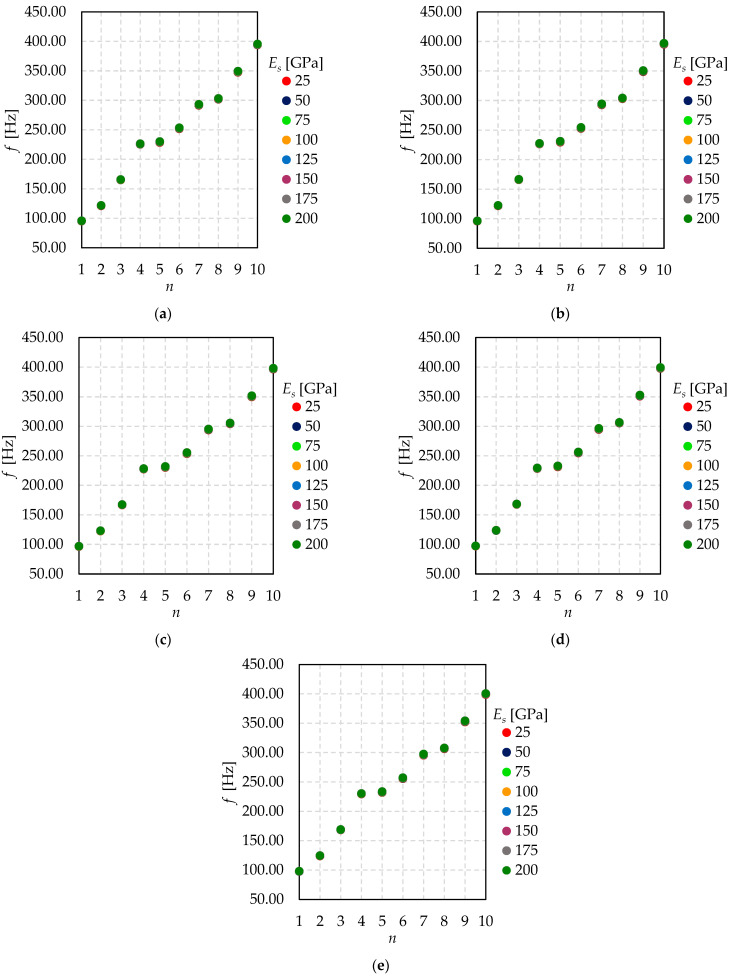
The relationship between natural frequency (*f*) and mode shape number (*n*) for different sealing Young’s modulus (*E_s_*)—(**a**) *E_p_* = 160 GPa; (**b**) *E_p_* = 170 GPa; (**c**) *E_p_* = 180 GPa; (**d**) *E_p_* = 190 GPa; (**e**) *E_p_* = 200 GPa.

Based on the results presented in Figure 16, there is hardly any difference in natural frequencies, considering all the examined Young’s modulus of the sealing. Analogously, it is nearly impossible to notice any significant difference in natural frequencies for all the investigated Young’s modulus of the pillars.

**Figure 17:**
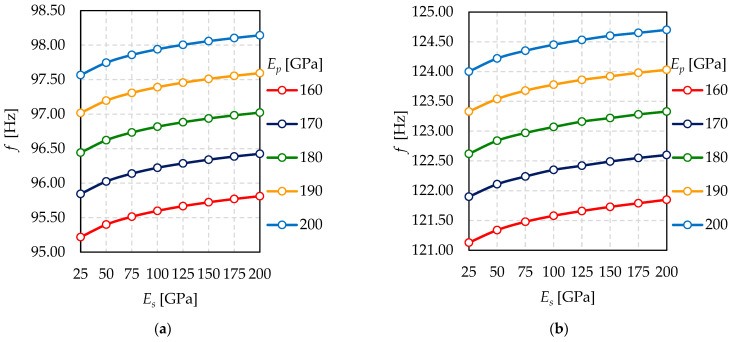
The relationship between natural frequency (*f*) and sealing Young’s modulus (*E_s_*) for different pillar Young’s modulus (*E_p_*)—(**a**) mode shape *n* = 1; (**b**) mode shape *n* = 2.

According to Figure 17, the greater Young’s modulus of the sealing, the greater the natural frequency, considering different Young’s modulus of the pillars. However, the differences in frequencies are up to 0.2%, compared with a 200% difference in the value of the sealing Young’s modulus.

Similarly, the greater Young’s modulus of the pillars, the greater natural frequency, considering different Young’s modulus of the sealing. Nonetheless, the differences in frequencies are up to 0.7%, compared with 6.3% difference in the value of the sealing Young’s modulus.

The influence of the pillars’ Young’s modulus on natural frequencies is decisively more significant than the influence of the sealing Young’s modulus.

Changes in material properties of support-pillars also have a more significant impact on dynamic characteristics than changes in Young’s modulus of sealing. As mentioned in Section 3.1.2, a more detailed analysis of these changes was made using the 3D model.

## 4. Conclusions and Future Research

In this study, the influence of different geometrical and material parameters on dynamic and static response of the VIG plates was investigated. In order to obtain appropriate values required for the comparison, different approaches for the vacuum glazing FEM model were considered and the most suitable were chosen. A proper numerical model will be an important tool for the future studies that concern VIG panels.

According to the results, the mechanical and geometrical properties of the support pillars are crucial considering not only the static response of the VIG plates, but also the dynamic one.

Considering the static analysis, despite pillars being located pretty close to each other, spacing between them cannot be larger due to the deflection of glass panes. The results obtained for the cases, in which the pillars spacing was constant, show that the greater displacements occur in the corners of the VIG plate, between the first and the second row of pillars. When verifying the load capacity of vacuum glazing, this area should be prudently considered. Nevertheless, getting rid of the first row of pillars near every edge seal led to a huge increase in deflection of the glass panes between the seal and the second row of pillars. Therefore, support pillars spacing must be carefully defined. Furthermore, it should be mentioned that pillars located in vacuum glazing are constantly exposed to sunlight, which can lead to their degradation over time. The above study shows that the violation of VIG structure leads to huge deflections, which in turn could lead to partial irreversible or complete destruction of these elements.

Another important aspect of the vacuum glazing is its dynamic response associated with out-of-phase vibrations. It applies to the glass panes due to the rapidly decreasing distance between them during the mentioned type of vibrations. The less spacing between the support pillars and consequently the stiffer the whole VIG plate, the greater the possibility for the aforementioned vibrations to occur. Due to the undesirability of this phenomenon, spacing between support pillars should be large enough.

In order to fulfil the above analysis, VIG plates are planned to be tested experimentally. They will be subjected to forced vibrations of specific frequencies. Going forward, there will be reviewed parameters such as vibration frequency, phase shift, internal dumping, etc. Having gathered data from the numerical and experimental analyses, it will be used as input data for artificial intelligence methods. The studies will be focused on neural networks (NN) and extreme gradient boosting (XGB) algorithms. Finally, the mechanical parameters of the support pillars hidden inside the analysed VIG plate will be received. To take advantage of the created numerical models, FEM thermal calculations are planned to be executed. Furthermore, conducting analyses related to the optimization of pillars’ distribution inside the VIG structure are considered. Fractal methods and genetic algorithms are planned to be used. This whole study will create an efficient tool for further research and optimization of material as well as geometrical parameters of vacuum glazing.

Future application of Artificial Intelligence methods aims to extend the possibilities of analysing vacuum glazing in existing buildings. VIG technology is gaining more and more popularity all around the world. This type of glazing, being constantly exposed to weather conditions such as sunlight or wind forces, is susceptible to degradation over time. Due to the importance of the pillars’ strength properties and the impossibility of testing them by disassembly, finding the optimal methods for testing their current strength in a non-destructive way will extend the scope of application of this type of window.

## Figures and Tables

**Table 1:** Variable parameters in the first category of analysis—geometrical parameters.

No	*h* (mm)	*x*_0_, *y*_0_ (mm)	*n_x_* (-)	*n_y_* (-)	*Φ* (mm)	No	*h* (mm)	*x*_0_, *y*_0_ (mm)	*n_x_* (-)	*n_y_* (-)	*Φ* (mm)
1	4	50	11	23	0.30	29	6	50	11	23	0.30
2	4	50	11	23	0.35	30	6	50	11	23	0.35
3	4	50	11	23	0.40	31	6	50	11	23	0.40
4	4	50	11	23	0.45	32	6	50	11	23	0.45
5	4	50	11	23	0.50	33	6	50	11	23	0.50
6	4	50	11	23	0.55	34	6	50	11	23	0.55
7	4	50	11	23	0.60	35	6	50	11	23	0.60
8	4	100	9	21	0.30	36	6	100	9	21	0.30
9	4	100	9	21	0.35	37	6	100	9	21	0.35
10	4	100	9	21	0.40	38	6	100	9	21	0.40
11	4	100	9	21	0.45	39	6	100	9	21	0.45
12	4	100	9	21	0.50	40	6	100	9	21	0.50
13	4	100	9	21	0.55	41	6	100	9	21	0.55
14	4	100	9	21	0.60	42	6	100	9	21	0.60
15	5	50	11	23	0.30	43	8	50	11	23	0.30
16	5	50	11	23	0.35	44	8	50	11	23	0.35
17	5	50	11	23	0.40	45	8	50	11	23	0.40
18	5	50	11	23	0.45	46	8	50	11	23	0.45
19	5	50	11	23	0.50	47	8	50	11	23	0.50
20	5	50	11	23	0.55	48	8	50	11	23	0.55
21	5	50	11	23	0.60	49	8	50	11	23	0.60
22	5	100	9	21	0.30	50	8	100	9	21	0.30
23	5	100	9	21	0.35	51	8	100	9	21	0.35
24	5	100	9	21	0.40	52	8	100	9	21	0.40
25	5	100	9	21	0.45	53	8	100	9	21	0.45
26	5	100	9	21	0.50	54	8	100	9	21	0.50
27	5	100	9	21	0.55	55	8	100	9	21	0.55
28	5	100	9	21	0.60	56	8	100	9	21	0.60

**Table 2:** Variable parameters in the second category of analysis—material parameters.

No	*E_p_* (GPa)	*E_s_* (GPa)	No	*E_p_* (GPa)	*E_s_* (GPa)	No	*E_p_* (GPa)	*E_s_* (GPa)
1	160	0.01	16	170	150	31	190	75
2	160	25	17	170	175	32	190	100
3	160	50	18	170	200	33	190	125
4	160	75	19	180	0.01	34	190	150
5	160	100	20	180	25	35	190	175
6	160	125	21	180	50	36	190	200
7	160	150	22	180	75	37	200	0.01
8	160	175	23	180	100	38	200	25
9	160	200	24	180	125	39	200	50
10	170	0.01	25	180	150	40	200	75
11	170	25	26	180	175	41	200	100
12	170	50	27	180	200	42	200	125
13	170	75	28	190	0.01	43	200	150
14	170	100	29	190	25	44	200	175
15	170	125	30	190	50	45	200	200

## Data Availability

The data presented in this study are available on request from the corresponding author.
